# Symptoms of maternal psychological distress during pregnancy: sex-specific effects for neonatal morbidity

**DOI:** 10.1515/jpm-2021-0340

**Published:** 2022-09-27

**Authors:** Sandra J. Weiss, Joseph W. Musana

**Affiliations:** Department of Community Health Systems, University of California, San Francisco, CA, USA; University of California, San Francisco, CA, USA; Department of Obstetrics & Gynaecology, Aga Khan University Hospital, Nairobi, Kenya

**Keywords:** gestational age, neonatal morbidity, pregnancy, psychological symptoms, sex differences

## Abstract

**Objectives:**

Maternal psychological distress during pregnancy has been associated with preterm birth. However, little is known about the relationship of a woman’s psychological symptoms during pregnancy to the infant’s morbidity at birth or any differential effects of these symptoms on female vs. male fetuses. Our research aims addressed these gaps.

**Methods:**

A total of 186 women were enrolled between 24 and 34 weeks gestation when demographic information was acquired and they completed the Brief Symptom Inventory to measure psychological distress. Data on gestational age at birth, fetal sex, and neonatal morbidity was extracted from the medical record. To control for their effects, obstetric complications were also identified. Multiple linear regressions were computed to examine the aims, including interaction terms to measure moderating effects of fetal sex.

**Results:**

Symptoms of maternal psychological distress were a significant predictor of neonatal morbidity but were not associated with gestational age. The interaction between symptom distress and fetal/infant sex was also significant for neonatal morbidity but not for gestational age. For boys, high levels of maternal symptom distress during pregnancy were associated with neonatal resuscitation, ventilatory assistance, and infection. Maternal distress was not associated with neonatal morbidity for girls.

**Conclusions:**

The male fetus may be more sensitive to effects of mothers’ psychological symptoms than the female fetus. Further research is needed to confirm our findings and identify potential biological mechanisms that may be responsible for these sex differences. Findings suggest the importance of symptom screening and early intervention to reduce maternal distress and risk of neonatal morbidity.

## Introduction

Pregnancy is a period of profound psycho-physiological transformation associated with potential emotional distress for a significant proportion of women. Psychological distress in this population primarily involves symptoms associated with depression and anxiety [1, 2], and often reflects high levels of perceived stress [3]. Approximately 10–25 percent of women worldwide suffer depressive symptoms during pregnancy while another 17–25 percent of women experience symptoms related to anxiety [4], [5], [6].

### Psychological distress and birth outcomes

In addition to the suffering it may cause for women, psychological distress during pregnancy has been linked to adverse birth outcomes. A number of studies report the impact of stress on outcomes such as preterm birth, low birth weight, intrauterine growth restriction, and small size for gestational age [7], [8], [9], [10], [11]. A systematic review by Grigoriadis et al. [12] found that prenatal depression was associated with preterm delivery, although the effects were modest. In addition, a recent review of research examining effects of prenatal anxiety on birth outcomes indicated that anxiety increased the odds for preterm birth, low birth weight, and being small for gestational age [13]. However, these findings are not consistent across studies, with a number reporting no relationship between maternal psychological distress and birth weight or preterm birth [14], [15], [16], [17].

It is also noteworthy that few studies have examined the relationship between maternal distress during pregnancy and actual neonatal morbidity. Most studies have only examined associations of distress with outcomes such as preterm birth or low birth weight. However, a few studies have found that antenatal depression is associated with greater newborn illness [18], with hypoxia, low apgar, and encephalopathy at birth [19], and with admission to intensive care units by infants [20], suggesting augmented severity of medical problems incurred by the neonate. In addition, prenatal life stress has been associated with worse neurological indicators for infants at birth [10]. Still, other studies have found no association between antenatal psychological distress and neonatal morbidity [13,21].

### Sex of the fetus as a moderator

Some of the inconsistency in findings regarding maternal distress and birth outcomes may be related to potentially different effects of maternal distress depending on sex of the fetus. Little attention has been given to the role of fetal sex as a potential moderator of maternal distress during pregnancy. Studies do suggest that, irrespective of maternal psychological distress, male fetuses are at greater risk of preterm birth than female fetuses [22], [23], [24], [25], [26] and have greater morbidity during the perinatal period [27, 28]. A few studies have assessed the effect of stressful environments that may induce psychological distress during pregnancy on differential outcome by sex. Two studies examined exposure to environmental stressors such as earthquakes and rocket blasts on birth weight and gestational age [29, 30]. Both studies found that female fetuses were more negatively impacted than males when their mothers were exposed to these stressors. Females showed significantly greater risk for preterm birth, earlier gestational age and small head circumference while males showed little if any effect. A third study reported that prenatal negative life events were associated with shorter gestational age, but for male infants only [31]. It is important to note that these studies evaluated the effect of exogenous stressors rather than symptoms of psychological distress that the woman may have experienced as a result of these environmental events. External stressors are experienced differently by various individuals, with some people feeling extensive distress from particular stressors while others may not.

We found only a few studies that examined sex differences in the relationship between actual psychological distress and birth outcomes. Two studies found a greater impact on female fetuses. Ae-Ngibise et al. [32] reported that female fetuses showed greater reduced birth length and weight as well as smaller head circumference than males when women were stressed. Kaitz et al. [33] assessed differential effects of maternal antenatal anxiety on infant birth weight for males vs. females, with females having lower weights than males when exposed to anxiety. However, birth weights remained in the normal range, with no adverse effect of anxiety on either sex. In contrast, two other studies found greater effects of prenatal distress on males. Walsh et al. [34] found that male fetuses, but not females, of mothers who were high in stress, anxiety and depression were born earlier and more likely preterm than mothers with minimal psychological distress. Similarly, Khashan and colleagues [35] found that high anxiety and depression among mothers during pregnancy increased the risk for male but not female fetuses to be small for gestational age at birth. Lastly, Edwards and Hans [36] found no evidence of sex differences in neonatal health problems associated with maternal depressive symptoms during pregnancy. Based on findings to date, the effect of women’s self-reported or perceived pregnancy distress on the male vs. female fetus remains very unclear.

### Purpose of the research

Our study sought to advance existing knowledge about the relationship of maternal distress during pregnancy to birth outcomes, with a particular focus on neonatal morbidity and potential differences in effects on the male vs. female fetus. Specific aims of the study were:

1) to examine the association of maternal psychological distress during pregnancy to infant gestational age and neonatal morbidity, and
2) to determine whether these relationships may differ based on sex of the fetus.

## Materials and methods

### Sample and recruitment

Because our outcomes of interest were gestational age and neonatal morbidity, it was important to recruit a sample of women who would have an adequate distribution on these variables. Women at risk of preterm birth during pregnancy do not always deliver prematurely but do yield a broad distribution on gestational age and morbidity of their infants. Women who were identified by their obstetrician or the clinic’s clinical research coordinator as being at risk for preterm birth were recruited from three clinics in the San Francisco Bay Area when they were between 24 and 34 weeks gestation. This time during pregnancy was selected because many of the risks for preterm birth are not detected until this gestational period, especially for women from underserved communities who may not have had ongoing prenatal care. For purposes of our study, criteria for being at risk for preterm labor included short interval between pregnancies, previous preterm birth, high blood pressure, cervical insufficiency or short cervix, uterine abnormality, fetal growth restriction, placenta previa, or preeclampsia. However, women were retained in the study whether they delivered preterm or not. Women were excluded if they did not speak English or Spanish or had cognitive impairments that prevented their informed consent or their completion of questionnaires. A research assistant approached women who were referred for potential participation and informed them of the study’s purpose and activities. The research was approved by the Institutional Review Board of the University of California, San Francisco.

### Measures

#### Self-report questionnaires

After consent to participate, women completed a demographic questionnaire and the Brief Symptom Inventory (BSI) [37]. The demographic questionnaire solicited basic information about the women, such as maternal age, income, race/ethnicity, and education. The BSI is a 53 item self-report inventory that assesses psychological symptoms such as depression and anxiety. It is a brief version of the Hopkins Symptom Checklist 90-R (SCL-90-R) [38]. Correlations between the BSI and SCL-R-90 are reported to range from 0.92 to 0.99 [37]. The Positive Symptom Distress Index (PSDI) of the BSI was used as the score in analyses of our aims. It measures intensity of symptoms experienced. The PSDI is calculated by summing the values of all symptoms that are endorsed divided by the symptom total. In addition to using the PSDI in analyses, we also split women into high (n=95, 51%) and low (n=91, 49%) distress groups based on the intensity of their symptoms.

Good internal consistency and test-retest reliability have been supported by several studies [39, 40]. Studies have also demonstrated the validity of the measure in accurately identifying distress in samples from various racial and ethnic backgrounds [41, 42]. There is the strongest psychometric support for use of scores from the total measure rather than individual subscales for depression, anxiety or other symptoms [43].

#### Measures based on medical record review

Using a structured template, research assistants reviewed medical records to identify: (a) specific medical complications experienced by women during the perinatal period, (b) infant gestational age, (c) medical or surgical problems incurred by infants during delivery or in the first month postnatal, and (d) a woman’s history of current or previous psychiatric diagnoses. These data were used to complete the Obstetrics Complication Scale (OCS) and the Perinatal Complications Scale (PCS) [44] and adjust for any significant covariates in our analyses.

##### Obstetric and perinatal complications

All items in the OCS and PCS are rated as present or not regarding a particular medical complication. Items are summed to yield a total score on each scale. The 41 items in the OCS covered common complications experienced by women such as diabetes, pre-eclampsia, genitourinary infections, or hypertension. The total scale on the OCS was used to control for maternal obstetric complications in testing of the aims. The 10 items in the PCS included problems such as respiratory distress, need for assisted ventilation, convulsions, non-infectious illness, and hyperbilirubinemia. The total number of problems identified on the PCS was used as a measure of neonatal morbidity. Content validity of these scales stemmed from a series of studies by a panel of expert clinicians [45]. Predictive and discriminant validity have been supported through association of the scale items with identified risk groups and varied clinical outcomes [46], [47], [48].

### Data analysis

Descriptive statistics were used to identify sample characteristics. Hierarchical multiple linear regression procedures were computed to analyze the research aims, with separate models for gestational age and neonatal morbidity. Scores for obstetric complications were entered at the 1st step to control for potential confounding effects. Psychological distress and fetal sex were entered at the 2nd step, with interaction terms for sex and distress entered at the 3rd step of the analyses. Regressions were also computed separately for girls and boys to examine more specific associations that might underlie significant interactions. Lastly, we performed analyses of covariance to identify potential differences in the prevalence of specific morbidities for infants whose mothers had high and low levels of psychological symptom distress. In these analyses, we controlled for obstetric complications. Statistical analyses were carried out with Stata version 16.

## Results

### Sample description

As shown in Table 1, women who participated in the study averaged 28 years of age, with a spread from 14 to 45. Thirty eight percent were European-American/White. Twenty percent were African-American/Black, and 32% were of Hispanic/Latina heritage. The remainder was from Asian and other backgrounds. Participants’ years of education ranged from no education to 21 years, with their average number being 12 (i.e. high school graduation). Women experienced a spectrum of complications during pregnancy, with the mean number being 11.2 out of a possible 20. Similarly, the intensity of women’s psychological distress showed a good distribution, with the median symptom distress of the group being in the moderate range (i.e. 2 in a range of 0–4).

**Table 1:** Maternal and neonatal characteristics for total sample and by sex.

Variable	All participants	Boys	Girls	p-Value for sex differences
	Mean (SD) Median (min–max)	Mean (SD) Median (min–max)	Mean (SD) Median (min–max)	
Mother’s age, years	28 (7) 30 (14–45)	28 (7) 30 (14–43)	29 (6) 30 (16–45)	0.44
Obstetric complications	11.2 (3.5) 11 (2–20)	10.9 (3.4) 10 (2–19)	11.6 (3.6) 11 (5–20)	0.22
Maternal symptoms of psychological distress	1.72 (0.92) 2.0 (0–4)	1.68 (0.82) 2.0 (0–4)	1.78 (1.82) 2.0 (0–4)	0.52
Neonatal morbidity	4.2 (2.3) 4 (0–10)	4.3 (2.3) 4.0 (0–10)	4.0 (2.2) 4.0 (0–9)	0.56
Gestational age, weeks	31.9 (3.5) 32 (23.5–40.0)	32 (3.1) 32 (25–39)	31.7 (3.9) 32 (23.5–40)	0.61

The sample included slightly more boys (56%) than girls (44%). Although the number of medical and surgical morbidities of infants spanned the full scope from 0 to 10, data indicate that the infants, as a group, had modest levels of morbidity (M=4.2). Their average gestational age was 31.9 weeks, ranging from 23.5 to 40.0 weeks. There were no significant differences between girls and boys in any of the maternal or infant variables being studied (see Table 1).

Only four women had an identified psychiatric diagnosis (either a Depressive or Anxiety Disorder) so we were not able to potentially include this variable in our regression models. However, the means for these four women did not appear to differ substantially from the larger sample on gestational age of their infants (30.3 weeks with diagnosis; 31.9 without diagnosis), or their infants’ neonatal morbidity (3.6 medical problems with diagnosis; 4.2 medical problems without diagnosis).

### Symptoms of psychological distress as a predictor of gestational age

For infants as a whole, there was no direct effect of psychological distress on gestational age nor was there any moderating effect of fetal sex on the relationship between psychological distress and gestational age. The beta coefficient for psychological distress was −0.088, p=0.214. The only predictor of gestational age for all infants combined was the number of obstetric complications (β=−0.317, p=0.0001; total model F=7.38 (df=3), p=0.001). However, as shown in Table 2, the gestational age of boys was affected more adversely by a mother’s complications during pregnancy than was gestational age of girls.

**Table 2:** Psychological distress as a predictor of gestational age for boys vs. girls, controlling for obstetric complications.

Variable	Beta coefficient (SE)	95% CI	p-Value
**Girls**			
Obstetric complications	−0.205 (0.12)	−0.462, 0.016	0.067
Psychological distress	−0.084 (0.42)	−1.164, 0.520	0.449

**Boys**			
Obstetric complications	−0.414 (0.08)	−0.553, −0.217	0.001
Psychological distress	−0.076 (0.35)	−0.976, 0.399	0.407

### Symptoms of psychological distress as a predictor of neonatal morbidity

**Figure 1:**
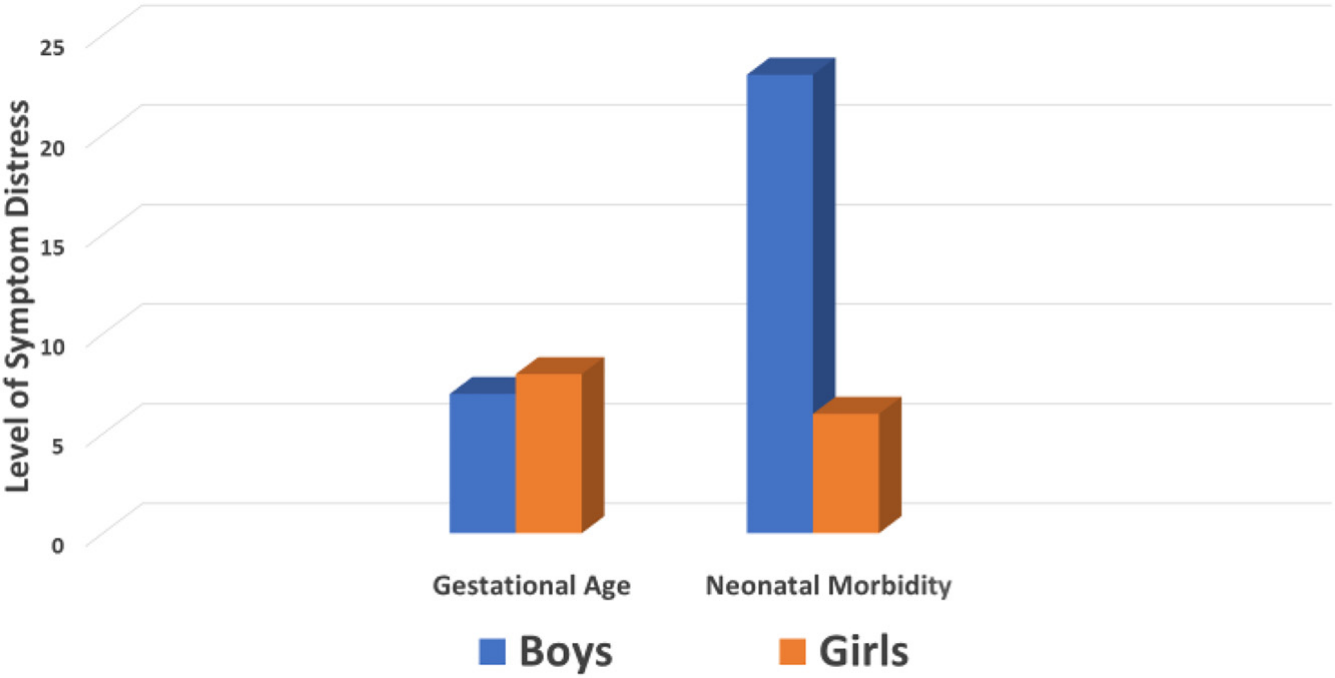
Sex-specific differences in the relationship of maternal psychological distress to gestational age and neonatal morbidity.

The regression model for neonatal morbidity indicated that psychological distress was a significant predictor of greater neonatal morbidity when all infants were combined (β=1.809, p=0.0001). However, the interaction term was also significant, indicating a moderating effect of fetal sex (β=−1.695, p=0.0001; total model F=16.325 [df=4], p=0.001). Sex-specific data in Table 3 shows that boys had greater neonatal morbidity when the intensity of their mother’s symptom distress was higher while there was no association between symptom distress and neonatal morbidity for girls. Sex differences in the strength of the beta coefficients for gestational age and neonatal morbidity are presented in Figure 1.

**Table 3:** Psychological distress as a predictor of neonatal morbidity for boys vs. girls, controlling for obstetric complications.

Variable	Beta coefficient (SE)	95% CI	p-Value
**Girls**			
Obstetric complications	0.135 (0.07)	−0.052, 0.216	0.231
Psychological distress	0.065 (0.24)	−0.333, 0.609	0.561
**Boys**			
Obstetric complications	0.153 (0.07)	−0.026, 0.236	0.116
Psychological distress	0.229 (0.27)	0.106, 1.18	0.019

**Figure 2:**
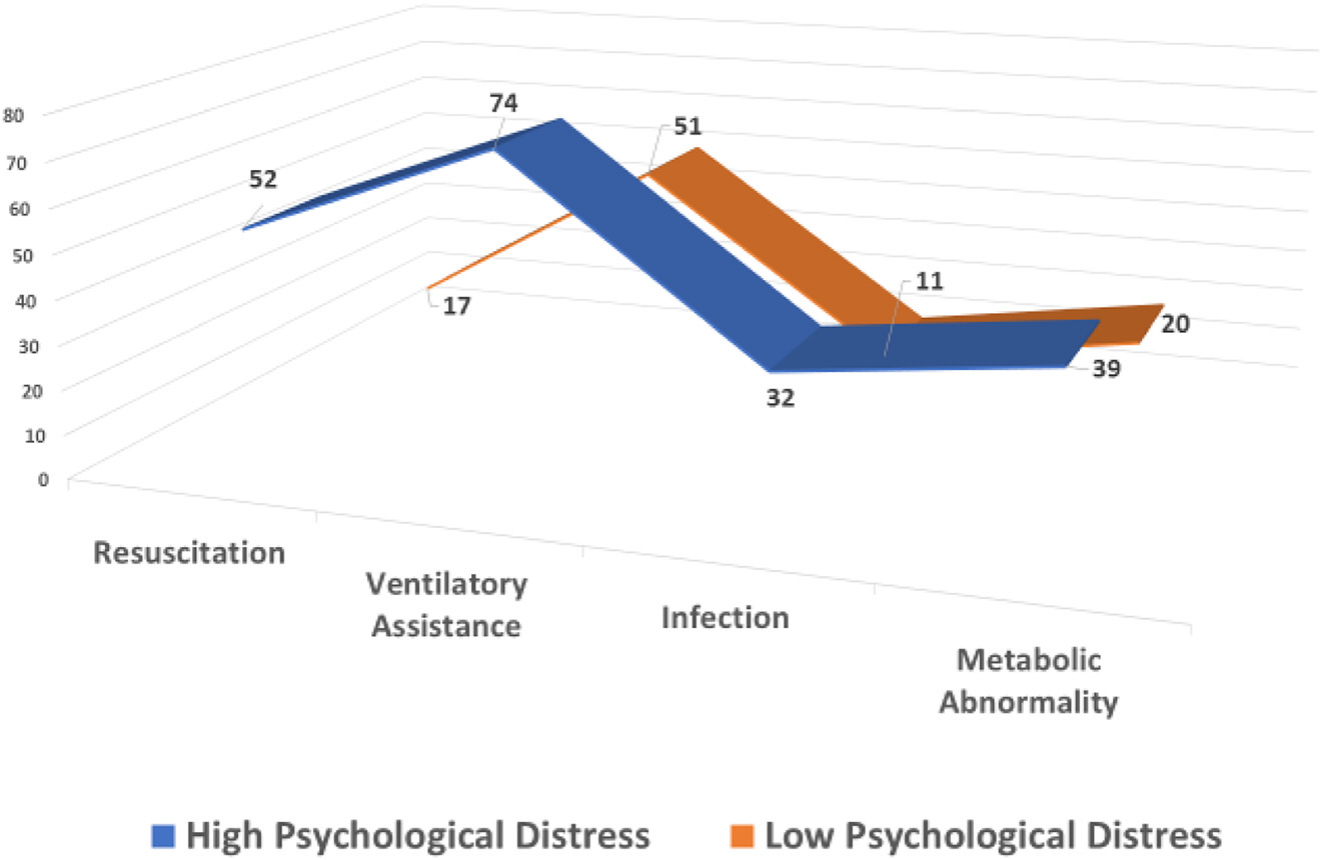
Mean values on key neonatal morbidities for boys whose mothers experienced high and low psychological distress.

After controlling for obstetric complications, analyses of covariance indicated significant differences between boys whose mothers had high and low psychological distress in three specific morbidities. The most significant of these was the need for resuscitation, with boys whose mothers had high levels of distress during pregnancy being more likely to require resuscitation at birth (F(1.64)=6.83, p=0.01). The presence of infection after birth was also higher in boys whose mothers had high levels of distress than those whose mothers had low symptom distress (F(1.64)=4.44, p=0.039). Lastly, the need for ventilatory assistance was significantly greater among boys whose mothers had high levels of symptom distress during pregnancy than those whose mothers had low symptom distress (F(1.64)=3.72, p=0.05). There was a trend toward a significant difference for metabolic abnormality (F(1.64)=2.84, p=0.08). Mean differences in these morbidity scores for boys whose mothers experienced high and low levels of symptom distress are highlighted in Figure 2. None of the morbidities showed significant differences between girl infants whose mothers had high and low symptom distress.

## Discussion

Findings indicate that the intensity of a woman’s distress from psychological symptoms during pregnancy was associated with severity of neonatal morbidity, but only for boys. However, symptoms of maternal psychological distress were not associated with gestational age of either boys or girls.

Our results for gestational age support the findings of two systematic reviews on prenatal depression [12, 49], indicating relationships of depression to the *occurrence* of preterm birth across some studies, but that most studies did not demonstrate differential effects of depression status related to the *actual length of gestation* itself. In concert with previous research, our results suggest that psychological distress may not have a differential impact on infants being delivered earlier vs. later preterm.

Instead, our findings indicate that women’s psychological symptoms during pregnancy may contribute more to the development of medical problems for the neonate than to shorter length of gestation. Other emerging evidence supports the potential impact that maternal psychological distress may have on the developing fetus, suggesting impairments in fetal brain biochemistry and brain growth [50, 51] as well as neonatal immunity [52, 53]. Such alterations have clear implications for neonatal morbidity, including the need for resuscitation and higher incidence of neonatal infections we found among infants whose mothers had more symptoms of psychological distress.

Of particular interest is our finding that the effects on morbidity were sex-specific. The morbidity of males was significantly associated with their mothers’ psychological symptoms while morbidity of females was not. To our knowledge, this study is the first to identify specific sex-linked morbidities that may be related to mothers’ prenatal psychological distress. A few studies have reported earlier gestational age [34] and smaller size for gestational age [35] among male infants but no effects on neonatal morbidity per se. Maternal psychological distress may interact in unique ways with sex hormones, the immune system, and the placenta of male vs. female fetuses, leading to different birth outcomes. In support of this premise, increased placental inflammatory lesions have been demonstrated in male but not female preterm deliveries [54], and mothers of male neonates born preterm (but not female) have demonstrated higher circulating pro-inflammatory cytokines and lower anti-inflammatory cytokines [55]. These findings may have particular relevance for the very robust association we found between higher pregnancy distress of mothers and the greater prevalence of infection in their male neonates. Results of previous research suggest that the female fetus is better protected than the male fetus from inflammatory processes that can compromise viability [56].

Other research suggests that male placentas may be more sensitive to acute stress while female placentas respond more to chronic stress [57]. However, the degree of acute vs. chronic symptom distress experienced by women in our study is not known. Based on their synthesis of research in the field, Sutherland and Brunwasser [58] propose that males are less adaptable to environmental challenges *in utero* in contrast to female fetuses who are more responsive to environmental stress signals and thus better able to adapt to prenatal insults.

Male placentas or fetuses may be more susceptible to pregnancy-related perturbations of the hypothalamic-pituitary –adrenal (HPA) axis, the autonomic nervous system or the immune system than females [59]. Each of these systems could be influenced by exposure to maternal symptoms of psychological distress. Research is needed to examine: (a) sex-specific up-regulation of stress hormones, inflammatory cytokines or catecholamines, and (b) sex-specific effects on gene expression and epigenetic modifications associated with maternal symptom distress during pregnancy. Effects of specific types of symptom distress should also be assessed to better understand any differential impact of depression, anxiety, or general perceived stress on neonatal outcomes.

### Limitations and strengths of the study

Limitations of this research should be considered when evaluating implications of the findings. Because our study included women who were identified as being at risk for preterm birth, the findings may not be generalizable to pregnant women in the population as a whole. It is possible that clinicians did not refer certain women experiencing psychological distress for study participation because of their concerns about the woman’s emotional fragility. Women experiencing greater distress may have also been more likely to decline participation. Such factors could contribute to selection bias. Our measure of neonatal morbidity assessed the overall number of medical problems incurred by an infant but did not weight them on severity or by specific class. In addition, our measure of psychological distress did not distinguish between different types of symptoms, such as depression or anxiety, nor did it distinguish between women with a mental health diagnosis and those without one.

However, our research has important strengths. Few studies have looked at the impact of maternal distress during pregnancy on neonatal morbidity incurred by the infant. Most studies have only examined associations with general classifications of preterm birth or low birth weight status. Because of their high risk status as a group of primarily preterm infants, our sample provided a good distribution on neonatal health problems. This increased our ability to detect potential effects of maternal distress on severity of neonatal morbidity and specific morbidities. This represents an innovative aspect of our research that can contribute to a more nuanced body of knowledge regarding adverse birth outcomes associated with maternal psychological distress. In addition, our research involved a very racially and ethnically diverse group of women. We also had an excellent distribution of our participants in terms of maternal age, education, and psychological distress, obstetric complications, gestational age and neonatal morbidity. These factors enhance the generalizability of our findings to a substantial degree.

## Conclusions

Results suggest that the intensity of women’s distress from psychological symptoms during pregnancy may have a greater effect on severity of neonatal morbidity than gestational age per se. In addition, the male fetus may be more vulnerable to the effects of maternal psychological distress than the female fetus. Further research is needed to confirm these findings and identify potential biological mechanisms that may be responsible for this sex difference.

Results of our research suggest that a reduction in a mother’s psychological symptoms during pregnancy could play an important role in reducing infant risk of greater neonatal morbidity, especially for boys. Until future studies elucidate mechanisms underlying specific effects of maternal psychological distress, it would be wise for clinicians to ensure that women are screened for symptoms of depression, anxiety and other mental health problems during pregnancy so that treatment can be provided when needed. In addition to potentially beneficial effects for their newborns, interventions for women are important for reducing their emotional suffering and helping them to manage both their pregnancy-related and general life stress.
